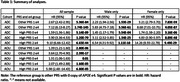# Using polygenic risk scores and *APOE* e4 to evaluate the risk for Alzheimer’s disease in European ancestry populations

**DOI:** 10.1002/alz.089048

**Published:** 2025-01-03

**Authors:** Dongbing Lai, Michael Zhang, Tatiana M. Foroud

**Affiliations:** ^1^ Indiana University School of Medicine, Indianapolis, IN USA

## Abstract

**Background:**

*APOE* e4 has been used to evaluate the risk for Alzheimer’s diseases (AD) but there exist other AD risk genes, and their effects can be collectively measured by polygenic risk scores (PRS). In this study, we sought to use both PRS (*APOE* excluded) and *APOE* e4 to evaluate the AD risk.

**Method:**

The discovery dataset was meta‐analysis of three large‐scale European ancestry AD GWAS (Kunkle et al, 2019, the UK Biobank, and the FinnGen consortium). SNPs within 500Kb from transcript starting and ending sites of *APOE* were excluded. PRS‐CS was used to calculate PRS. Target datasets were European ancestry samples from NIA Alzheimer’s disease centers (ADC, 2,413 cases and 3,423 controls) and All of Us research program (AOU, 1,177 cases and 60,607 controls). Participants having age at onset (cases) or age at the last interview (controls) <60 were excluded. The prevalence of AD were higher in ADC (41.35%) and lower in AOU (1.91%) than those in general populations; therefore, we combined ADC and AOU samples to approximate the PRS distribution in general populations. Then we dichotomized PRS as high (highest 10%) and other (the remaining 90%) based on the PRS distribution of combined sample. Cox proportional hazard model was used to test the effects of dichotomized PRS and e4 genotypes (0, 1, and 2 copies of e4 alleles) by adjusting for sex. Additionally, we performed sex stratified analyses in ADC only as numbers of high PRS and e4/e4 carriers in AOU in either sex were <5.

**Result:**

Results are summarized in Table 1. We used those having other PRS and no e4 as the reference group. In both ADC and AOU, high PRS or e4 were significantly associated with the AD risk (PRS hazard ratios (HRs): 1.31‐1.74, P‐values≤0.01; e4 HRs: 1.39‐8.68, P‐values≤2.80E‐06) but having both substantially increased the AD risk (HRs: 2.07‐14.26, P‐values≤6.80E‐05). In ADC, both high PRS and e4 had larger effects in females than in males.

**Conclusion:**

The effects of PRS were modest and cannot be used alone to evaluate the AD risk; however, PRS can potentially be used with *APOE* e4 to evaluate the AD risk.